# New malignancies after squamous cell carcinoma and melanomas: a population-based study from Norway

**DOI:** 10.1186/1471-2407-14-210

**Published:** 2014-03-19

**Authors:** Trude E Robsahm, Margaret R Karagas, Judy R Rees, Astri Syse

**Affiliations:** 1 Cancer Registry of Norway, PB 5313 Majorstuen, N-0304 Oslo, Norway; 2 Department of Community and Family Medicine, Geisel School of Medicine, Section for Epidemiology and Biostatistics, Dartmouth Medical School, HB 7927, One Medical Center Drive, Lebanon, NH 03756, USA; 3 Statisics Norway, PB 8131 Dep, 0033 Oslo, Norway

**Keywords:** Malignant Melanoma, Squamous Cell Carcinoma, Second cancer, Population-based, Sociodemographic factors, Family

## Abstract

**Background:**

Skin cancer survivors experience an increased risk for subsequent malignancies but the associated risk factors are poorly understood. This study examined the risk of a new primary cancer following an initial skin cancer and assessed risk factors associated with second primary cancers.

**Methods:**

All invasive cutaneous malignant melanomas (CMM, N = 28 069) and squamous cell carcinomas (SCC, N = 24 620) diagnosed in Norway during 1955–2008 were included. Rates of new primary cancers in skin cancer survivors were compared to rates of primary malignancies in the general population using standardized incidence ratios (SIR). Discrete-time logistic regression models were applied to individual-level data to estimate cancer risk among those with and without a prior skin cancer, accounting for residential region, education, income, parenthood, marital status and parental cancer status, using a 20% random sample of the entire Norwegian population as reference. Further analyses of the skin cancer cohort were undertaken to determine risk factors related to subsequent cancers.

**Results:**

During follow-up, 9608 new primary cancers occurred after an initial skin cancer. SIR analyses showed 50% and 90% increased risks for any cancer after CMM and SCC, respectively (p < 0.01). The logistic regression model suggested even stronger increase after SCC (130%). The highest risk was seen for subsequent skin cancers, but several non-skin cancers were also diagnosed in excess: oral, lung, colon, breast, prostate, thyroid, leukemia, lymphoma and central nervous system. Factors that were associated with increased risk of subsequent cancers include male sex, older age, lower residential latitude, being married and low education and income. Parental cancer did not increase the risk of a subsequent cancer after SCC, but was a significant predictor among younger CMM survivors.

**Conclusions:**

Our results provide information on shared environmental and genetic risk factors for first and later cancers and may help to identify individuals at high risk for subsequent cancers, which will be important as skin cancer incidence continues to rise.

## Background

Cutaneous squamous cell carcinoma (SCC) and malignant melanoma (CMM) are among the most prevalent [1] and rapidly increasing malignancies in white populations worldwide [2]. In Norway, the incidence of these cancers has increased sevenfold during the last fifty years. In 2010, SCC and CMM represented more than 10% of all new cancer cases in Norway [3]. The major environmental risk factor for all skin cancers is ultraviolet radiation (UVR) from sunlight; consequently, there is a distinct north–south gradient in skin cancer risk [4]. The type of UVR exposure (e.g. intermittent versus accumulated) appears to differ for SCC and CMM [5]. In addition, individual characteristics such as skin type, eye and hair color, nevi and immune suppression are important predictors of individual susceptibility to skin cancer [6,7]. High socioeconomic status is also associated with CMM [8].

SCC is a highly curable disease, and the most recent five-year relative survival rates in Norway are 89% and 93% for men and women respectively [3]. Although CMM carries a higher mortality rate, the 5-year survival rate is still high (77% and 89% for men and women, respectively) [3], due to early diagnosis and successful treatment. During the last fifteen years, several studies have suggested that a history of skin cancer increases the risk for a new primary cancer, both of the skin and other sites [9-30]. Many of these studies were based on cancer registry data, with high quality case ascertainment but lacking data on individual risk factors that might explain this. Other studies have benefitted from individual exposure data collected using questionnaires, but these tended to be smaller and have less rigorous confirmation of skin and subsequent cancers. A reduced risk of new malignancies after skin cancer has also been reported for some cancer sites [31-37], which some argue is due to the potentially protective effect of high levels of vitamin D that result from UVR exposure [38].

From the Nordic countries, Sweden, Finland and Denmark have reported their risks of second cancers after skin cancer [12-14,18,28,29], whereas Norway has not previously reported on these risks. Therefore, we used the Norwegian Cancer Registry data to describe the risk of a new primary cancer following SCC or CMM, and to further assess whether sociodemographic factors or cancer family history were associated with risk of subsequent cancers after skin cancer. Identifying those at greatest risk for subsequent cancers may benefit future public health initiatives by targeting interventions appropriately in the prevention and/or early detection of subsequent malignancies.

## Methods

### Study population

Our study cohort included all persons with a histologically verified invasive cutaneous malignant melanoma (N = 28 069) or squamous cell carcinoma (N = 24 620) diagnosed as a first primary cancer in the period 1955–2008 (Table 1). The *Cancer Registry of Norway* has registered all cancer diagnoses nationwide from 1953 onwards (not including basal cell carcinoma of skin). Mandatory reporting from multiple independent sources ensures the collection of complete and high quality data [39]. Available information includes date and type of primary skin cancer diagnosis, stage, and anatomical location (head/neck, trunk, arm, leg/foot or other sites) as well as similar data on subsequent cancers. To distinguish new primary cancers from recurrences, the Cancer Registry uses histological information and medical record review; if the histology reports are similar, at least four months must have passed since the initial cancer diagnosis for a later case to be defined as a new primary cancer and clinical records must be consistent with a new primary according to the cancer registry standard [39].

**Table 1:** Descriptive features of the cohort of persons with a first primary cutaneous malignant melanoma (CMM) or squamous cell carcinoma (SCC) at time of the primary skin cancer diagnosis

	**CMM**				**SCC**			
	**Women**	**%**	**Men**	**%**	**Women**	**%**	**Men**	**%**
**Age at 1**^**st **^**diagnosis**								
20-40	3249	21.5	1943	15.0	248	2.3	270	2.0
40-59	5435	35.9	4908	38.0	1201	11.0	1492	10.9
≥ 60	6452	42.6	6082	47.0	9474	86.7	11 935	87.1
**Age (median)**	55.6		57.4		75.4		73.2	
**Anatomical site**								
Head/neck	2148	14.2	2025	15.7	6560	60.0	8809	64.3
Trunk	4353	28.8	7044	54.4	1659	15.2	2071	15.1
Arm	2346	15.5	1188	9.2	1103	10.1	1281	9.4
Leg/foot	5633	37.2	1786	13.8	1060	9.7	737	5.4
Other sites	656	4.3	890	6.9	541	5.0	799	5.8
**Residential region**								
South	10 875	71.9	9270	71.7	7878	72.1	9537	69.6
Mid	2998	19.8	2525	19.5	2235	20.5	2877	21.0
North	1263	8.3	1138	8.8	810	7.4	1283	9.4
**Educational level**								
≤ Primary school	496	3.3	617	4.8	420	3.8	653	4.8
Secondary school	5199	34.4	3446	26.6	5678	52.0	5847	42.7
Some college	4545	30.0	3271	25.3	3106	28.4	3359	24.5
≥ College degree	4896	32.3	5599	43.3	1719	15.8	3830	28.0
**Marital status**								
Not married	6270	41.4	3515	27.2	6981	63.9	4641	33.9
Married	8866	58.6	9418	72.8	3942	36.1	9056	66.1
**Parental status**								
No children	3875	25.6	2989	23.1	3855	35.3	4226	30.9
Children	11 261	74.4	9944	76.9	7068	64.7	9471	69.1
**Annual income**								
No or missing	6467	42.7	4118	31.8	8930	81.7	9523	69.5
$ 1-9999	2573	17.0	1639	12.7	801	7.3	1609	11.8
$ 10 000–19 999	1712	11.3	1283	9.9	320	3.0	567	4.1
$ 20 000–39 999	2326	15.4	2166	16.8	462	4.2	782	5.7
$ ≥ 40 000	2058	13.6	3727	28.8	410	3.8	1216	8.9
**Duration of follow-up (years)**								
0.0-2.0	2551	16.9	3050	23.6	2642	24.2	3491	25.5
2.1-5.0	2938	19.4	3235	25.0	3019	27.6	3875	28.3
5.1-10.0	3044	20.1	2610	20.2	2658	24.3	3427	25.0
10.1-15.0	2261	14.9	1577	12.2	1398	12.8	1655	12.1
15.1-20.0	1720	11.4	1088	8.4	648	6.0	690	5.0
20.1-25.0	1109	7.3	615	4.8	289	2.6	313	2.3
≥ 25.1	1513	10.0	758	5.8	269	2.5	246	1.8
**Total number**	15 136	100	12 933	100	10 923	100	13 697	100
No subsequent cancer	13 078	86.4	10 995	85.0	9030	82.7	9978	72.9
Any subsequent cancer	2058	13.6	1938	15.0	1893	17.3	3719	27.1

The *Norwegian Population Register,* the *Norwegian Education Register* and the *Norwegian Directorate of Taxes* provided information for the cases as well as the general population on individual level characteristics such as date of birth, emigration and death, residential region, attained educational level (1960, 1970, and yearly from 1980 onwards), income (annual from 1966 onwards), parity, and marital status. The data were linked through unique personal identification numbers assigned to every individual residing in Norway from 1960 onwards. Permission to match the data was provided by the National Data Inspectorate in Norway after ethical review of this study by the Norwegian Board of Medical Ethics.

For our study, we obtained individual-level data on all residents during the period 1955–2008 (N = 5.3 million). We used all skin cancer cases and a 20% random sample of the entire population for comparison (N = 1.1 million). Follow-up for skin cancer cases began on the date of diagnosis. For the general population, follow-up began either at age 20 or age in 1955 if greater than 20 at that time. Age and calendar period were included as time-varying covariates, 20–39, 40–59 and ≥ 60 years and 1955–64, 1965–74, 1975–84, 1985–94, 1995–2004 and 2005 and above, respectively. As the majority of Norwegians have completed their education by age 25 and having children by age 35 for the time period under consideration, we used educational level and parental status at end of follow-up in our models. For residential region, marital status and income, we modeled status at the start of follow-up. The average follow-up time was 10.1 years for skin cancer survivors, and 30.6 years for the general population.

We examined the associations between an initial skin cancer and new primary cancers of the skin as well as mouth/pharynx, lung, breast, prostate, thyroid, leukemia and lymphomas, which are the sites with the most evidence of an association in earlier studies [10-12,14-16,19,21,23,25-30]. Exploratory analyses were also undertaken to examine the risk of second cancer at other common sites.

We examined the risk of second cancers by age at diagnosis because young age at onset of the initial cancer may indicate a genetic predisposition. We examined residence by latitude (South, Mid and North, as previously described [40]) because the association between skin cancer and subsequent malignancies is hypothesized to be strongest further from the equator [21]. To account for detection bias attributable to increased attention to cancer symptoms after a skin cancer diagnosis, we also examined the short- and long-term risks of subsequent cancer after diagnosis of a primary skin cancer.

We obtained information on parental cancer diagnoses for the skin cancer survivors who had identified parents (i.e. were alive in 1960). Overall, we were able to link 60% of the skin cancer survivors with their mother and 55% with their father. In general, the linkage rate decreased with increasing age at diagnosis, and we thus performed analyses stratified by year of birth (all, birth year > 1930 and birth year > 1950).

### Statistical analyses

Three types of analyses were conducted. First, we used standardized incidence ratios (SIR) to compare rates of subsequent malignancies in skin cancer survivors with rates of first primary cancer in subgroups of the general population of comparable age, sex and calendar time. The person-years at risk and the observed number of cancer cases were counted by sex within 5-year age groups (20–24, …, 80–84, 85+), and 5-year calendar periods (1955–59, …, 2000–04), except for the last period which ran from 2005–08. The expected number of cancer cases was calculated by multiplying the number of person-years in each age group and calendar period in the skin cancer cohort by the corresponding cancer rates in the general Norwegian population. The 95% confidence interval (CI) for the SIR was based on the assumption that the observed number of cases was Poisson-distributed and the expected number was non-stochastic. The analyses were performed using Stata 12 [41].

Second, a discrete-time logistic regression model was used to assess the importance of individual-level risk factors for the risk of a new primary cancer after skin cancer versus a first primary malignancy. Series of twelve-month observations were created for each individual. Individuals were followed from start of follow-up to a first primary malignancy (in a 20% random selection of the general population) or a subsequent malignancy (in skin cancer survivors). Individuals were followed to 2008, unless death, emigration or a malignancy occurred before this time. The follow-up time was categorized as 0–2 years, 3–5 years, 6–10 years, 11–15 years, 16–20 years, 21–25 years or more than 25 years. Categorizations of the covariates are shown in Table 2. Interaction terms between a primary skin cancer diagnosis and the various sociodemographic features were included to assess possible effect modification. Based on these results, stratified analyses were undertaken on variables for which effect modification appeared to be present.

**Table 2:** Odds ratios (OR) and 95% confidence intervals (CI) from a fully saturated discrete-time logistic regression model, comparing the risk of a first cancer in the general population with that of a second cancer in skin cancer survivors

	**CMM**		**SCC**	
	**Events/Pyr**^**1**^	**OR (95% CI)**	**Events/Pyr**	**OR (95% CI)**
**Primary skin cancer**				
No	143 978/31.0 mill	1.00 ref	144 179/31.0 mill	1.00 ref
Yes	3662/288 823	1.53 (1.48,1.59)	5612/175 641	2.30 (2.22,2.37)
**Sex**				
Female	72 768/15.8 mill	1.00 ref	72 319/15.7 mill	1.00 ref
Male	75 206/15.4 mill	1.28 (1.26,1.29)	77 472/15.4 mill	1.31 (1.29,1.32)
**Age**^**2**^				
20-40 years	6447/12.0 mill	1.00 ref	6509/12.0 mill	1.00 ref
40-59 years	30 795/10.6 mill	5.37 (5.22,5.52)	30 241/10.5 mill	5.28 (5.14,5.43)
≥ 60 years	110 732/8.6 mill	20.58 (20.04,21.14)	113 041/8.6 mill	20.34 (19.80,20.9)
**Residential region**^**3**^				
North	21 277/6.0 mill	1.00 ref	21 323/6.0 mill	1.00 ref
Mid	34 586/7.2 mill	1.30 (1.27,1.32)	34878/7.2 mill	1.30 (1.28,1.32)
South	92 111/18.0 mill	1.33 (1.31,1.35)	93 590/17.9 mill	1.35 (1.33,1.37)
**Calendar period**^**2**^				
< 1965	8477/4.3 mill	1.00 ref	8354/4.3 mill	1.00 ref
1965-1974	20 484/5.4 mill	2.54 (2.47,2.61)	20 867/5.4 mill	2.62 (2.56,2.6)
1975-1984	27 276/5.9 mill	3.63 (3.54,3.73)	27 573/5.9 mill	3.73 (3.63,3.83)
1985-1994	33 071/6.3 mill	4.28 (4.17,4.40)	33 817/6.3 mill	4.45 (4.34,4.57)
1995-2004	40 142/6.6 mill	5.33 (5.19,5.47)	40 241/6.6 mill	5.41 (5.27,5.56)
≥ 2005	18 524/2.7 mill	5.99 (5.82,6.17)	18 939/2.6 mill	6.22 (6.04,6.40)
**Educational level**^**4**^				
≤ Primary school	15 926/1.9 mill	1.00 ref	16 141/1.9 mill	1.00 ref
Secondary school	67 042/11.8 mill	0.32 (0.31,0.33)	67 915/11.8 mill	0.32 (0.31,0.33)
Some college	35 646/7.0 mill	0.30 (0.29,0.31)	36 079/7.0 mill	0.30 (0.29,0.30)
≥ College degree	29 360/10.5 mill	0.30 (0.29,0.31)	29 656/10.4 mill	0.30 (0.30,0.31)
**Marital status**^**3**^				
Not married	47 085/15.7 mill	1.00 ref	47 881/15.7 mill	1.00 ref
Married	100 889/15.5 mill	1.25 (1.24,1.27)	101 910/15.4 mill	1.27 (1.25,1.28)
**Parental status**^**4**^				
No children	138 424/28.9 mill	1.00 ref	139 451/28.9 mill	1.00 ref
Children	9550/2.3 mill	0.95 (0.93,0.97)	10 340/2.2 mill	0.96 (0.94,0.98)
**Income (annual)**^**3**^				
$ 0^5^	60 395/9.1 mill	1.00 ref	62 871/9.1 mill	1.00 ref
$ 80–9 999	40 528/10.7 mill	0.92 (0.90,0.93)	40 556/10.7 mill	0.91 (0.90,0.92)
$ 10 000–19 999	34 425/8.7 mill	0.80 (0.79,0.82)	34 394/8.7 mill	0.80 (0.78,0.80)
$ 20 000–39 999	11 582/2.6 mill	0.78 (0.77,0.80)	11 140/2.5 mill	0.76 (0.74,0.78)
$ ≥ 40 000	1044/153 034	0.82 (0.77,0.87)	830/122 013	0.78 (0.73,0.84)

^1^Number of events (new cancers) per person-year during follow-up. ^2^Time-varying covariates (yearly). ^3^At start of follow-up. ^4^At end of follow-up. ^5^Includes also those with missing income data.

**Figure 1:**
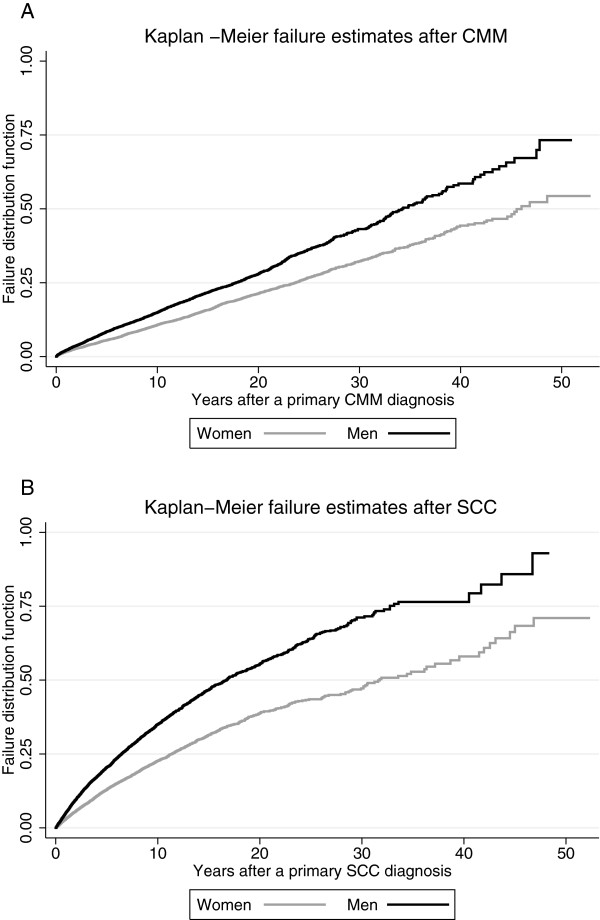
Kaplan-Meier plots of the timing of a second malignancy for men and women after a primary diagnosis of cutaneous malignant melanoma (A) and a primary diagnosis of cutaneous squamous cell carcinoma (B).

Third, internal analyses were undertaken to examine whether cancer-related and sociodemographic factors influence the risk of subsequent malignancies within the cohort of skin cancer survivors. The analyses explored the possible impact of the anatomic site of the primary skin cancer and history of parental cancer. All the discrete-time models were run using the PROC LOGISTIC procedure in SAS 9.2 [42]. Time to subsequent malignancies in men and women was explored using Kaplan-Meier plots, separately for CMM (Figure 1A) and SCC (Figure 1B) survivors, using Stata 12 [41]. The statistical significance level was set at 5%.

## Results

Overall, 3996 and 5612 subsequent cancers occurred, respectively, among CMM and SCC survivors during follow-up. The characteristics of individuals diagnosed with skin cancer are displayed in Table 1. CMM patients were diagnosed at an earlier age, were better educated, had higher incomes, were more frequently married and had children than SCC patients.

### Standard incidence ratios (SIR)

We found 50% and 90% increases in the risks of subsequent cancer after an initial CMM and a SCC diagnosis, respectively (Tables 3, 4). The strongest risks were observed for subsequent skin cancers, and in particular for skin cancers of the same histology as the index cancer. Increased risks were also observed for several non-skin cancers (thyroid, breast, prostate, leukemia and lymphomas) after both types of skin cancer. After CMM, a 30% increase in risk of central nervous system (CNS) tumors was observed and in men we observed a decreased risk of lung cancer. After SCC, more than a threefold and a sixfold risk increase was observed for mouth/pharynx and salivary gland tumors respectively, as well as a nearly 50% risk increase for lung cancer. Further, the risks of pancreatic and colon cancer were significantly elevated in both sexes, but in women we found a decreased risk of cancers of urinary organs.

**Table 3:** Standard incidence ratios (SIR) with 95% confidence intervals (CI) for a second malignancy by cancer site in men and women with a history of cutaneous malignant melanoma (CMM), adjusted for age and calendar period

		**Women (N = 15 184**^**1**^**)**		**Men (N = 12 972**^**1**^**)**	
**ICD-10**	**Cancer site**^**2**^	**O**^**3**^	**SIR (95% CI)**	**O**^**3**^	**SIR (95% CI)**
C00-14	Mouth, pharynx	14	0.77 (0.46,1.30)	33	1.11 (0.79,1.56)
C18	Colon	163	1.02 (0.88,1.19)	134	1.16 (0.98,1.37)
C19-21	Rectum	57	0.87 (0.67,1.13)	62	0.93 (0.72,1.19)
C25	Pancreas	46	1.00 (0.76,1.33)	39	1.06 (0.77,1.45)
C33,34,38	Lung	109	1.12 (0.93,1.35)	119	0.72 (0.60,0.87)
C50	Breast	377	1.24 (1.12,1.37)	-	-
C50-58	Female organs	588	1.18 (1.09,1.28)	-	-
C61	Prostate	-	-	445	1.26 (1.15,1.38)
C64-68	Urinary organs	68	0.93 (0.73,1.18)	139	0.99 (0.84,1.17)
C70-72	CNS^4^	70	1.39 (1.09,1.75)	41	1.31 (0.98,1.78)
C73	Thyroid	19	1.27 (0.81,1.99)	13	2.78 (1.61,4.80)
C81-85	Lymphoma	56	1.29 (0.99,1.68)	60	1.56 (1.20,1.99)
C91-95	Leukemia	72	1.28 (1.01,1.60)	64	1.22 (0.87,1.43)
C00-96^5^	Other sites	169	1.15 (0.99,1.34)	176	1.07 (0.92,1.24)
C43	CMM	438	7.59 (6.91,8.33)	411	9.44 (8.57,10.4)
C44	SCC	197	3.12 (2.71,3.59)	204	3.11 (2.71,3.56)
C00-96	All sites	2066	1.52 (1.46,1.59)	1943	1.49 (1.43,1.60)
C00-96^6^	All, exc. skin	1431	1.16 (1.11,1.23)	1328	1.12 (1.06,1.18)
C00-96^7^	All, exc. CMM	1628	1.26 (1.20,1.32)	1532	1.25 (1.19,1.31)

^1^The number of cancer cases in the SIR-analyses differs slightly from that used in the logistic regression analyses as the data were extracted on different dates from the Cancer Registry of Norway. ^2^The cancer sites are not mutually exclusive. ^3^Observed number of cases. ^4^Central nervous system tumors. ^5^Includes ICD-10 codes C15-17, C22-24, C26, C30-32, C37, C39-41, C45-49, C60, C62-63, C69, C74-76, C80, C88, C90. ^6^All sites except C43 and C44. ^7^All sites except C43.

**Table 4:** Standard incidence ratios (SIR) and 95% confidence intervals (CI) for a second malignancy by cancer site in men and women with a history of cutaneous squamous cell carcinoma (SCC), adjusted for age and calendar period

		**Women (N = 10 965**^**1**^**)**		**Men (N = 13 748**^**1**^**)**	
**ICD-10**	**Cancer site**^**2**^	**O**^**3**^	**SIR (95% CI)**	**O**^**3**^	**SIR (95% CI)**
C00-14^4^	Mouth, pharynx	38	3.03 (2.20,4.16)	114	3.23 (2.69,3.87)
C07-08	Salivary glands	11	6.69 (3.71,12.1)	17	6.19 (3.85,9.95)
C18	Colon	176	1.25 (1.08,1.45)	197	1.09 (0.95,1.25)
C19-21	Rectum	45	0.86 (0.64,1.14)	96	0.98 (0.80,1.20)
C25	Pancreas	45	1.03 (0.77,1.38)	82	1.39 (1.12,1.73)
C33,34,38	Lung	82	1.46 (1.17,1.81)	303	1.49 (1.32,1.66)
C50	Breast	204	1.15 (1.00,1.32)	-	-
C50-58	Female organs	320	1.11 (0.99,1.24)	-	-
C61	Prostate	-	-	650	1.14 (1.06,1.23)
C64-68	Urinary organs	42	0.69 (0.51,0.94)	222	1.08 (0.94,1.23)
C70-72	CNS^5^	17	0.65 (0.40,1.04)	32	1.10 (0.78,1.56)
C73	Thyroid	12	7.30 (4.15,12.8)	3	0.60 (0.19,1.85)
C81-85	Lymphoma	64	2.14 (1.67,2.74)	113	2.41 (2.00,2.90)
C91-95	Leukemia	65	1.33 (1.04,1.69)	150	1.70 (1.45,1.99)
C00-96^6^	Other sites	183	1.13 (0.98,1.31)	371	1.37 (1.23,1.51)
C43	CMM	95	3.09 (2.52,3.78)	122	2.76 (2.31,3.29)
C44	SCC	715	9.88 (9.18,10,6)	1249	10.1 (9.51,10.6)
C00-96	All sites	1898	1.89 (1.81,1.98)	3725	1.91 (1.84,1.96)
C00-96^7^	All, exc. skin	1088	1.22 (1.15,1.30)	2354	1.33 (1.28,1.39)
C00-96^8^	All, exc. SCC	1183	1.23 (1.16,1.30)	2476	1.31 (1.25,1.36)

^1^The number of cancer cases in the SIR-analyses differs slightly from that used in the logistic regression analyses as the data were extracted on different dates from the Cancer Registry of Norway. ^2^The cancer sites are not mutually exclusive. ^3^Observed number of cases. ^4^Includes ICD-10 codes C00-06 and C09-14. ^5^Central nervous system tumors. ^6^Includes ICD-10 codes C15-17, C22-24, C26, C30-32, C37, C39-41, C45-49, C60, C62-63, C69, C74-76, C80, C88, C90. ^7^All sites except C43 and C44. ^8^All sites except C44.

### Logistic regression models

As several cancer forms are associated with socioeconomic status, site-specific analyses controlling for sociodemographic and -economic risk factors were conducted. Results were fairly similar to those observed in the SIR analyses (Table 5), e.g., for the risk of breast cancer after either CMM or SCC. However, the model resulted in higher risk estimates for leukemia, lymphoma and colon cancer after CMM or SCC and for cancers of urinary organs after SCC. For prostate cancer there was a lower estimate after CMM but higher after SCC.

**Table 5:** **Odds ratios (OR) and 95% confidence intervals (CI) of subsequent cancer by cancer site for cohorts with a primary cutaneous malignant melanoma (CMM) or cutaneous squamous cell carcinoma (SCC) relative to that of the general population, adjusted for covariates shown in Table** 2^**1**^

		**CMM**		**SCC**	
**ICD-10**	**Cancer site**^**2**^	**Events**^**3**^	**OR (95% CI)**	**Events**	**OR (95% CI)**
C00-14^4^	Oral cavity	2768	0.96 (0.68,1.34)	2876	2.70 (2.19,3.34)
C07-08	Salivary glands	257	0.62 (0.14,2.74)	291	8.89 (5.29,14.93)
C18	Colon	13 281	1.28 (1.12,1.46)	13 190	1.62 (1.43,1.82)
C19-21	Rectum	6696	1.15 (0.93,1.42)	6768	1.10 (0.91,1.33)
C25	Pancreas	4551	1.14 (0.89,1.45)	4590	1.56 (1.27,1.91)
C33,34,38	Lung	12 347	0.72 (0.62,0.84)	12 613	1.23 (1.09,1.36)
C50	Breast	17 275	1.21 (1.07,1.36)	16 956	1.16 (1.01,1.34)
C50-58	Female organs	29 775	1.11 (1.02,1.22)	29 349	1.08 (0.96,1.21)
C61	Prostate	17 398	1.17 (1.02,1.34)	17 797	1.28 (1.14,1.43)
C64-68	Urinary organs	10 607	1.00 (0.85,1.18)	10 787	1.18 (1.03,1.37)
C70-72	CNS^5^	4348	1.73 (1.39,2.15)	4149	1.07 (0.79,1.44)
C73	Thyroid	1437	1.75 (1.19,2.56)	1435	1.27 (0.75,2.16)
C81-85	Lymphoma	4672	1.42 (1.14,1.76)	4678	2.78 (2.33,3.32)
C91-95	Leukemia	6096	1.63 (1.33,1.99)	6162	2.15 (1.83,2.53)
C00-96^6^	Other sites	21 969	1.27 (1.13,1.43)	22 373	1.67 (1.51,1.84)
C43	CMM	6363	7.99 (7.19,8.88)	5827	3.45 (2.94,4.05)
C44	SCC	5382	3.31 (2.86,3.82)	6906	14.4 (13.2,15.7)
C00-96	All sites	147 974	1.53 (1.48,1.59)	149 791	2.30 (2.22,2.37)
C00-96^7^	All, exc. skin	136 229	1.16 (1.11,1.21)	137 058	1.51 (1.46,1.57)

^1^The number of cancer cases in the SIR-analyses differs slightly from that used in the logistic regression analyses as the data were extracted on different dates from the Cancer Registry of Norway. The number of cases are nevertheless quite similar (CMM: SIR 28 156 vs 28 069, events 4009 vs 3996 and SCC: SIR 24 713 vs 24 620, event 5623 vs 5612). ^2^The cancer sites are mutually exclusive. ^3^Observed number of events in a 20% general population sample and the cohort of skin cancer survivors. ^4^Includes ICD-10 codes C00-06 and C09-14. ^5^Central nervous system tumors. ^6^Includes ICD-10 codes 150–152, 155, 156, 158–161, 164, 178, 179, 192, and 195–199. ^7^All sites except C43 and C44.

Table 2 shows estimates from a fully saturated discrete-time logistic regression model, comparing the risk of a first cancer in the general population with that of a second cancer in skin cancer survivors. Compared to the general population, the cancer risk was approximately 50% and 130% higher after an initial CMM and SCC, respectively. An increased cancer risk was associated with male sex, older age, lower residential latitude, recent calendar time, primary education, low income and being married.

In the comparison between CMM survivors and the general population, interactions were observed for educational attainment, attained age and calendar period (p_interaction_ < 0.01). In stratified analyses, the risk of a subsequent cancer was higher for CMM survivors with an education at college or university level (Odds ratio (OR) 1.65, CI 1.57-1.74) than for those with a lower education (OR 1.34, CI 1.26-1.42). Similarly, a CMM diagnosis before the age of 60 significantly increased the risk of a subsequent cancer (OR 2.59, CI 2.41-2.79) compared to those older than 60 years (OR 1.43, CI 1.37-1.50). The interaction term between calendar year as a linear variable and CMM diagnosis suggested an increase in risk with more recent calendar time. However, splitting the sample at year 1990 did not identify appreciable differences (≤ 1990: OR 1.57, CI 1.50-1.64 vs. > 1990: OR 1.64, CI 1.52-1.77).

For SCC survivors in comparison to the general population, interactions were found for parental status, educational attainment, attained age and calendar period (p_interaction_ < 0.01). Risk for a subsequent cancer was higher for SCC survivors with at least one child (OR 2.64, CI 2.48-2.80) compared to those without children (OR 2.26, CI 2.15-2.37). Further, a higher risk of subsequent cancer was observed for SCC survivors with an education at college or university level (OR 2.49, CI 2.38-2.62) compared to those with a lower education (OR 1.97, CI 1.89-2.06). SCC diagnosis before the age of 60 was associated with a higher risk of subsequent cancer (OR 5.54, CI 4.88-6.29) compared to those older than 60 years (OR 2.07, CI 2.00-2.15). Risk of subsequent cancer was also higher after 1990 (OR 2.52, CI 2.42-2.62) than prior to this (OR 2.29, CI 2.15-2.42).

#### Risk of subsequent cancer among skin cancer survivors

The results for the internal comparisons were quite similar for the two subgroups of skin cancer survivors and they confirmed the findings from the external analyses (Table 6). No clear difference in the overall risk of subsequent cancer was found across the anatomical sites of the primary skin cancer, after CMM or SCC (data not shown). This was true for all cancer sites, skin included.

**Table 6:** Odd ratios (OR) and 95% confidence intervals (CI) from a fully saturated discrete-time logistic regression model examining the risk for a subsequent cancer diagnosis within the cohorts with a primary cutaneous malignant melanoma (CMM) or squamous cell carcinoma (SCC)

	**CMM**		**SCC**	
	**Events/Pyr**^**1**^	**OR (95% CI)**	**Events/Pyr**	**OR (95% CI)**
**Sex**				
Female	2058/173 679	1.00 ref	1893/81 108	1.00 ref
Male	1938/115 144	1.51 (1.41,1.63)	3719/94 533	1.90 (1.79,2.01)
**Age**^**2**^				
< 40 years	80/31 862	1.00 ref	19/3020	1.00 ref
40-59 years	807/106 669	2.89 (2.29,3.65)	222//17 362	2.28 (1.42,3.66)
≥ 60 years	3109/147 183	6.67 (5.31,8.39)	5371/155 259	4.62 (2.93,7.29)
**Residential region**^**3**^				
North	275/23 927	1.00 ref	419/15 325	1.00 ref
Mid	703/54 955	1.09 (0.95,1.26)	1124/37 786	1.09 (0.98,1.23)
South	3018/209 941	1.20 (1.06,1.36)	4069/122 530	1.20 (1.08,1.33)
**Calendar period**^**2**^				
< 1965	6/2959	1.00 ref	35/4411	1.00 ref
1965-1974	71/13 448	3.01 (1.30,6.98)	168/10 542	2.51 (1.73,3.64)
1975-1984	276/34 380	4.92 (2.15,11.24)	438/22 580	3.61 (2.50,5.20)
1985-1994	913/69 763	7.80 (3.43,17.34)	1281/40 225	6.46 (4.52,9.24)
1995-2004	1707/112 983	8.88 (3.91,20.17)	2364/64 765	7.76 (5.44,11.1)
≥ 2004	1023/55 290	10.52 (4.63,23.9)	1326/33 118	8.73 (6.10,12.5)
**Educational level**^**4**^				
≤ Primary	63/5193	1.00 ref	178/6203	1.00 ref
Secondary	1285/85 407	0.80 (0.61,1.04)	2514/82 333	0.60 (0.50,0.71)
Some college	1293/85 733	0.79 (0.60,1.05)	1577/46 489	0.60 (0.50,0.72)
≥ College degree	1355/112 490	0.80 (0.61,1.05)	1343/40 616	0.61 (0.51,0.74)
**Marital status**^**3**^				
Not married	1089/84 671	1.00 ref	2161/72 265	1.00 ref
Married	2907/204 152	1.02 (0.94,1.10)	3451/103 376	1.01 (0.95,1.08)
**Parental status**^**4**^				
No children	881/68 530	1.00 ref	1692/56 884	1.00 ref
Children	3115/220 293	0.91 (0.84,0.99)	3920/118 757	0.93 (0.87,0.99)
**Income (annual)**^**3**^				
$ 0^5^	1586/81 524	1.00 ref	3982/113 773	1.00 ref
$ 80–9 999	679/57 594	0.83 (0.75,0.91)	715/24 518	0.85 (0.78,0.92)
$ 10 000–19 999	514/50 340	0.68 (0.61,0.75)	300/12 099	0.76 (0.67,0.85)
$ 20 000–39 999	713/57 131	0.75 (0.68,0.82)	334/13 692	0.65 (0.58,0.74)
$ ≥ 40 000	504/42 234	0.65 (0.58,0.73)	281/11 559	0.59 (0.51,0.67)

^1^Number of events (new cancers) per person-year during follow-up. ^2^Time-varying covariates (yearly). ^3^At start of follow-up. ^4^At end of follow-up. ^5^Includes also those with missing income data.

### Parental cancer

The risk for a new primary cancer after an initial CMM was not associated with a history of cancer in their mother (OR 1.05, CI 0.90-1.23), father (OR 0.95, CI 0.81-1.11) or either parent (OR 1.00, CI 0.88-1.13), for individuals born after 1930. When we restricted the analyses to those with complete parental linkage (individuals born after 1950), the respective estimates were elevated; risks of cancer after CMM in individuals with a history of cancer in their mother, father or either parent were 1.39 (CI 1.09-1.78), 1.38 (CI 1.09-1.76) and 1.52 (CI 1.21-1.92) respectively. For SCC survivors, the estimates associated with parental cancer were not significantly elevated in the overall or restricted analyses (data not shown).

### Time since first primary cancer

The risk of subsequent cancer after an initial skin cancer increased with time and the risk excess was greater for men than for women (Figures 1A and B). Whereas the increase appeared almost linear for CMM survivors, it rose in a non-linear manner for SCC survivors.

## Discussion

The risk of a new primary cancer was higher after an initial skin cancer compared to the risk of a first primary cancer in the general Norwegian population, using multiple approaches. The highest risk was found for a subsequent skin cancer, but several non-skin cancers were also diagnosed in excess including oral, colon, lung, breast, prostate, urinary organs, CNS, thyroid, leukemia and lymphomas. Many of the associations we observed have been reported previously, but we were able to account for a number of sociodemographic factors, and show that the risks remained elevated. Among skin cancer survivors an elevated risk of subsequent cancer was associated with male sex, older age, lower residential latitude, primary education only, low income and being married and the risk increased over the study period. Having children was associated with a slightly decreased cancer risk. Having a parent with a history of cancer increased the risk for a new primary cancer after CMM if diagnosed before age 60.

Individual risk of skin cancer is determined by UVR and host susceptibility [43]. These are the likely explanations for the increased risk of skin and perhaps lip cancer after a CMM or SCC diagnosis, but medical surveillance after the diagnosis may also be important. However, the reasons why individuals with a history of skin cancer have a higher risk of non-cutaneous malignancies, than those without a skin cancer history, are less obvious.

As in previous reports [9,10,14-16,18,19,21-23,25-30,36], we also observed that a history of skin cancer increased the risk of leukemia and lymphoma. Although the mechanism for such an association is not fully understood, several mechanisms have been suggested. First, UVR impairs the immune system, both locally in the skin and systemically [44-46]. Moreover, immunodeficiency as a result of the disease itself [27,47,48], genetic factors [44] or previous treatment with radiation or chemotherapy [49,50], are all potentially shared risk factors.

We found a history of SCC to be related to an increased risk of lung and mouth/pharynx cancers which is consistent with several previous observations [9,12,16,19-21,23,24,26-30,51]. Although UVR has been suggested to play a role also for these cancer types, similar relationships were not observed after CMM. The most reasonable explanation for the increased risk of lung and oral cancer in SCC survivors may be related to tobacco use, which also has been suggested as a risk factor for SCC [29,51]. Moreover, a primary SCC was associated with an increased risk of cancer in urinary organs that also might be related to tobacco. While we have no information on tobacco use, prior studies that were able to control for tobacco use still observed an increased risk of lung [20,30], oral and kidney cancers [30]. Thus, it is possible that the relationship observed between SCC and cancers of mouth/pharynx, lung, and urinary organs lies elsewhere.

The risk of thyroid cancer was significantly increased, both after an initial CMM (both sexes) and SCC (women only), also consistent with previous findings [11,21,25-27,31]. Common risk factors such as genetic or environmental exposure have been suggested to explain this association [25]. A cancer diagnosis may also increase future health vigilance, which may result in increased detection of new malignancies. Physical examination for CMM includes lymph node palpation in the thyroid area and we cannot exclude the possibility of such surveillance bias. Unfortunately, our numbers were too small to enable a detailed analysis of the development of thyroid cancer with time from a primary skin cancer diagnosis.

With respect to the elevated risk of CNS tumors, we cannot exclude the possibility that the second primary cancer, reported by the clinicians as a CNS tumor, could in some patients be a metastasis from the primary CMM. As CMM but not SCC is likely to spread to the brain, this may explain why no such association was observed between SCC and risk of CNS tumors.

In line with previous studies [10,15,21,25,27,33,34,52], an initial skin cancer diagnosis was found to be associated with an increased risk of colon, breast and prostate cancer. For breast cancer, the relationship was most pronounced after a primary diagnosis of CMM. Shared hormonal mechanisms for CMM and breast cancer have been proposed, although the epidemiological evidence of the role of hormones in melanoma etiology is inconsistent [52]. All these three cancer forms, as well as skin cancer, are associated with high socioeconomic status, and shared risk factors linked to socioeconomic status may account for the positive relationship, including frequent screening activity [25,34]. For CMM survivors this is somewhat confirmed by the lowering of the risk estimates for breast and prostate cancer once socioeconomic factors were taken into account. In contrast, for SCC survivors, the risk estimates for these three cancers rose when accounting for socioeconomic factors.

### Sociodemographic factors

Several cancer diseases are strongly associated with socioeconomic resources [53] and socioeconomic factors have been suggested to represent mediators by which environmental risk factors vary within a population. In the present study we observed the highest cancer risk in the lowest educational and income levels, and it is likely that this may be attributed to shared adverse lifestyle factors (i.e. smoking habits, alcohol intake, sun protection, diet). The Norwegian health care system intends to ensure equal health care for all inhabitants, independent of socioeconomic level. Nevertheless, individuals who belong to a lower social class tend to have lower health vigilance and participation in surveys and screening programs to minimize the burden of cancer [54,55]. Therefore, we cannot exclude the possibility that our results, at least partly, might be influenced by such mechanisms.

The high risk observed in the elderly may be due to changed sun exposure habits and increased outdoor activities after retirement [56], but there may also be an effect of an age-related diminished immune response [57,58]. Analyses stratified by age, on the other hand, showed that compared to persons in the general population, the risk of a second primary cancer was greatest for the youngest skin cancer survivors, particularly for SCC survivors. A possible explanation might be a genetic susceptibility to skin cancer, which is associated with young age at diagnosis [59], and such susceptibility may also increase the risk for subsequent cancers. Further, the stronger effect of young age seen for SCC survivors may be due to the different patterns of UVR exposure associated with the two skin cancer types (intermittent/accumulated) and to behavior after diagnosis. A CMM diagnosis may, because of its lethal potential, lead to a more sun-protective behavior compared to a SCC diagnosis.

Moreover, steep increases in skin cancer rates over time has been suggested to reflect increased UVR doses [56], and this may in part explain the observed increase of subsequent cancers by calendar time.

The status of being married was associated with an increased risk of subsequent cancer after skin cancer, while having children slightly decreased the risk. Having a family influences the lifestyle in several ways, as they may help ensure more optimal medical surveillance as well as reinforce better health behaviors after diagnosis (i.e. smoking habits, alcohol intake, sun protection, diet, screening attendance) [60], which may cause these relationships.

### Parental cancer

Young age at onset of the initial cancer may be an indication of a genetic predisposition [61]. Contrary to previously reported work [21,62,63], having a parent with cancer did not generally increase the risk of being diagnosed with a subsequent malignancy. However, analyses restricted to individuals born after 1950, showed a near 50% increase in risk when either parent had a history of cancer. Disentangling the effect of age and parental cancer is, however, not possible due to data limitations.

### Time since first primary cancer

An increased health awareness or close follow-up by the health care personnel may result in earlier diagnoses of new cancers in CMM and SCC survivors compared to that of the general cancer-free population [15,55]. Nevertheless, it is unlikely that this can fully explain the observed elevated cancer risk. Higher risk was observed in men than in women, and due to gender difference in health vigilance, we would expect the opposite as women more often interact with health care personnel [64]. The nonlinear increase for SCC survivors may help explain why the interaction terms between a skin cancer diagnosis and calendar year were statistically significant whereas the stratified analyses with a cut-point set arbitrarily in 1990 yielded overlapping confidence intervals for CMM, but not for SCC.

### Vitamin D

UVR promotes vitamin D synthesis and vitamin D is demonstrated to regulate several genes involved in cancer processes and is hypothesized to inhibit cancer development [38]. Inverse relationships between a history of skin cancer, as a proxy of high vitamin D levels, and risk of subsequent cancers support this hypothesis [31,32,34-37], but the vitamin D hypothesis is not supported by the large number of studies that report increased risks of second primary cancers after skin cancer [9-30,33], and neither by the present results. We found the highest risk of subsequent cancer in the South region, which has the highest UVR dose in Norway. Recently, a study including four cohorts from different regions of Norway observed a slightly higher mean level of serum vitamin D in the cohort comprising residents from the southern part of the country [65]. Therefore, low levels of vitamin D might not explain our findings, although we cannot exclude the possibility that individuals with a history of skin cancer have insufficient vitamin D-levels due to changed sun-exposure habits after diagnosis. Results from a Danish study, however, counters this hypothesis as it demonstrates that previous CMM patients not are more cautious sun bathers [66]. We can also not exclude that other effects of UVR exposure, such as immunodeficiency, may play a role.

### Limitations and strengths

The study has several obvious strengths including the long time-span, covering more than 50 years of follow-up, and the large study population, covering the entire Norwegian population. Several cancers were diagnosed in excess, but the highest risk was found for a subsequent skin cancer, particularly of similar type. General practitioners and dermatologists interacting with skin cancer survivors should be aware of this in their surveillance and follow-up.

Individual-level information on some potential confounding variables was available and was found to influence the risk estimates. Less adjusted models are thus likely to over- or underestimate the real risk increase in skin cancer survivors, depending on the direction of the influence of excluded variables. A limitation of the study is the lack of information on parental cancer in older skin cancer survivors. Further, the use of a 20% sample resulted in larger standard errors and thus wider confidence intervals than would have been obtained if we had included the whole population. This is particularly relevant for the site-specific analyses of less common cancer forms, but nevertheless unlikely to have impacted our estimated significantly. Another limitation is the lack of behavioral risk factors such as smoking habits, sun exposure habits, body mass index (BMI), physical activity etc., which would have given more direct information on behavioral changes that may minimize the risk for a second cancer. Lastly, as we chose to primarily focus on socioeconomic factors at time of diagnosis, we are unable to account for changes in these over the life course. Such analyses would shed light on the relevance of developments in socioeconomic characteristics that have taken place in Norway as well as in other industrialized countries over the last 50 years. Nonetheless, there is little reason to believe that the development has been much different for skin cancer survivors compared to the general population given that we adjusted for age, calendar period and follow-up time.

## Conclusions

Heightened public awareness may be important to prevent subsequent cancers in skin cancer survivors as the skin cancer incidence rates continue to rise. Of particular relevance to general practitioners and dermatologists dealing with skin cancer survivors, is the need for enhanced surveillance for a new skin cancer. Further, our results provide information on shared risk factors for first and later cancers that may help in identifying individuals at high risk for subsequent cancers, and for whom particular attention ought to be directed.

## Abbreviations

CMM: Cutaneous malignant melanoma; SCC: Squamous cell carcinoma; UVR: Ultraviolet radiation; CNS: Central nervous system; CI: Confidence interval; OR: Odds ratio; N: Number; SIR: Standardized incidence ratio; O: Observed number of cancer cases; N/A: Not applicable; Pyr: Person-years; BMI: Body mass index (kg/m^2^).

## Competing interests

The authors declare that they have no competing interest.

## Authors’ contributions

All named authors have met the criteria for authorship. All the authors made substantial contributions to conception and design and with interpretation of the results. The statistical analyses were performed by TER and AS, who also drafted the manuscript, which was critically revised by MRK and JRR. All authors ensure that the paper represent honest work and are able to verify the validity of the results reported. All authors read and approved the final manuscript.

## Authors’ information

TER holds a PhD in cancer epidemiology and is a senior researcher at the Cancer Registry of Norway. Her research focuses on lifestyle variables (physical activity, UVR, vitamin D) and cancer risk and survival. MRK is Professor of Community and Family Medicine in Epidemiology at Dartmouth Medical School and co-directs the Epidemiology and Chemoprevention program at Norris Cotton Cancer Center. Her research focuses on the etiology and prevention of human cancers. JRR is a physician epidemiologist who directs the New Hampshire State cancer registry and conducts research related to disease surveillance, vitamin D and clinical trial methodology. AS holds a PhD in public health and is a senior researcher at Statistics Norway in the Research Department. Her focus is concentrated on cancer survivorship, mortality, health, and health behaviors.

## Pre-publication history

The pre-publication history for this paper can be accessed here:

http://www.biomedcentral.com/1471-2407/14/210/prepub
